# The influence of cognitive value and role perception on community residents’ willingness to participate in age-friendly renovations

**DOI:** 10.3389/fpsyg.2026.1922438

**Published:** 2026-08-18

**Authors:** Yu Xiang, Ying Guan, Haoran Huang, Ru Meng, Linghao Rui, Muwei Zhu, Xinru Huang

**Affiliations:** 1 School of Management, Xuzhou Medical University, Xuzhou, China; 2 Department of Medical Insurance, Xuzhou Cancer Hospital, Xuzhou, Jiangsu, China; 3 Institution of Chinese Health Modernization, Xuzhou Medical University, Xuzhou, Jiangsu, China

**Keywords:** age-friendly renovations, cognitive value, community residents, mediating effect, participation willingness, role perception

## Abstract

**Objective:**

This study aims to examine how cognitive value and role perception influence residents’ willingness to participate in age-friendly community renovations, and to test a dual mediation model in which community attachment and institutional trust serve as parallel mediators.

**Methods:**

A cross-sectional survey design was employed, with data collected from 595 residents across several representative communities in a city in Jiangsu Province, China. Data were collected via a self-administered questionnaire and analyzed using SPSS 26.0 and the PROCESS macro. Multiple linear regression and Bootstrapping methods were applied to test the hypothesized relationships.

**Results:**

Significant positive correlations were found among cognitive value, role perception, community attachment, institutional trust, and participation willingness. Multiple linear regression indicated that cognitive value, role perception, community attachment, and institutional trust significantly predicted participation willingness (*F* = 25.81, *P* < 0.001, *R*^2^ = 0.284). Furthermore, community attachment and institutional trust served as significant mediators in the relationships between cognitive value and participation willingness and role perception and participation willingness, with indirect effect values of 0.108 (95% Bootstrap CI: 0.057, 0.158) and 0.145 (95% Bootstrap CI: 0.093, 0.201), respectively.

**Conclusion:**

The findings suggest that policymakers prioritize enhancing residents’ cognitive value and role perception while simultaneously strengthening community attachment and institutional trust, thereby effectively stimulating participation willingness and promoting the sustainable development of age-friendly community renovations. Such strategies satisfy residents’ psychological needs for meaning, belongingness, and security, facilitating policy goal internalization and achieving synergy between external guidance and internal motivation.

## Introduction

In the late 1990s, the concept of community care was formally incorporated into the aging policy agenda, marking the elevation of aging in place from grassroots initiatives to the national strategy (Means and Smith, 1998). Age-friendly renovations, as the key measure to address population aging, draws its principles from international concepts of accessibility and universal design (Mace, 1985). Through China’s policy implementation, it has been systematically developed into a home-centered retrofitting model, rapidly gaining widespread adoption in recent years under national policy initiatives. With rapid urbanization and social changes, many countries have begun to renovate old communities (Luo et al., 2022). China’s age-friendly renovations exhibit distinct characteristics. Unlike other government-led public initiatives, these projects require extensive resident participation in decision-making processes (You and Yang, 2025). Within the Chinese context, old communities refer to residential areas constructed before the year 2000, which typically suffer from inadequate management and maintenance, insufficient municipal support, and lacking community service facilities (Liu et al., 2024a). By 2024, China had completed renovations in over 240,000 aging communities, benefiting more than 40 million households and 110 million residents. This indicates that the age-friendly renovations of communities has become the key strategy for ensuring sustainable growth in Chinese cities (Zheng et al., 2023).

China’s community-based age-friendly renovations are government-led and partially government-funded (Huang et al., 2023). Nevertheless, as the direct beneficiaries of these renovations, community residents must also play an active role in the process. Regarding resident participation willingness, scholars have conducted exploratory research with notable outcomes. Zhang et al. (2021) using old residential communities in Hengshui City as a case study, found that residents’ willingness to participate in renovations stems from rational choices driven by material or spiritual benefits. Guo et al. (2019) highlighted the importance of perceived fairness in shaping residents’ willingness to participate in renovations. Researchers also identified the quality of public services as a key driver of resident engagement in community activities (Xiong and Zhou, 2021). Li and Wang (2021) examined the influence of emotional network within communities on residents’ willingness to participate in renovations, using the example of elevator installations in old residential areas in Guangzhou. Residents’ willingness to participate in age-friendly renovations is constrained by multiple factors, encompassing both rational calculations of personal interests and emotional, non-rational influences such as internal community social relationships and perceptions of fairness.

Previous studies have separately confirmed the associations between cognitive value, role perception, and participation willingness, as well as between community attachment, institutional trust, and participation willingness (Kant and Vertinsky, 2022; Li et al., 2022; Zhang et al., 2025). However, few studies have integrated psychological variables (value and role) and contextual perception variables (attachment and trust) within a single analytical framework, let alone examined their potential dual mediating pathways in the specific domain of community age-friendly renovations. Therefore, this study aims to construct a theoretical model to investigate how cognitive value and role perception influence community residents’ willingness to participate in age-friendly renovations, with a particular focus on the mediating mechanisms of community attachment and institutional trust.

## Literature review

### Cognitive value, role perception, and community residents’ participation willingness

Community resident participation serves as an important foundation for the effective governance and sustainable operation of age-friendly renovation projects. Unlike traditional government-led public infrastructure construction, age-friendly renovations involve multiple stages including resident demand expression, scheme consultation, process supervision, and subsequent maintenance. Whether residents are willing to participate depends not only on external policy supply but is also influenced by individual psychological cognition and awareness of social responsibility. Existing studies have shown that residents’ participation willingness is essentially a behavioral tendency formed based on individuals’ evaluation of behavioral outcomes and their recognition of their own social roles (You and Yang, 2025).

Cognitive value refers to an individual’s comprehensive evaluation of the functional value, social value, and long-term benefits that may arise from a certain behavior. In the field of public affairs participation, value cognition is considered an important psychological factor influencing individual participation decisions. You and Yang (2025), through a questionnaire survey of community residents, found that perceived value plays a partial mediating role between residents’ social capital and participation willingness, and that individuals typically form behavioral judgments by weighing behavioral benefits against costs. Similarly, Xu et al. (2024), based on survey data from farming households in Jiangxi Province, confirmed that functional value, emotional value, and cognitive value all have significant positive effects on participation willingness, with functional value exerting the largest effect. For age-friendly renovations, residents’ value cognition of renovation projects is reflected not only in the improvement of the current living environment but also in long-term benefits such as future elderly care security, community safety, and the reduction of family caregiving pressure. When residents fully recognize the positive significance of age-friendly renovations for both individuals and community development, they are more likely to form positive attitudes and further generate participation willingness.

The Theory of Planned Behavior (TPB) points out that behavioral willingness is primarily influenced by attitudes toward the behavior, subjective norms, and perceived behavioral control (Li et al., 2024). Among these, individuals’ positive evaluation of behavioral outcomes serves as an important foundation for the formation of behavioral attitudes. Therefore, the more fully residents recognize the value of age-friendly renovations, the stronger their positive evaluation of participation behaviors will be, ultimately leading to higher participation willingness.

Roles serve as the link between individuals and society, representing the most common and active mediating factor (Xi, 2010). Role perception constitutes the core of the role concept, reflecting individuals’ understanding of the responsibilities and functions they should undertake within the social system (Liu et al., 2018). In the context of community governance, residents’ participation behaviors are not merely policy responses but are rooted in their deep-seated recognition of the responsibilities they bear as community members. Zhang et al. (2025) demonstrated that perceived value exerts a significant direct positive influence on participation willingness. Li and Wang (2021) further demonstrated that the emotional networks and role cognition within communities jointly affect residents’ participation decisions regarding collective affairs. Age-friendly renovations are not only related to the direct interests of elderly residents but also involve the collective optimization of the entire community’s living environment; residents are not passive policy recipients but rather participating subjects in community governance. Accordingly, the stronger residents’ role perception is, the stronger their identification with participation as their due responsibility becomes, thereby leading to higher participation willingness.

Based on the above analysis, this study proposes that: cognitive value and role perception are positively correlated with residents’ participation willingness.

### The mediating role of community attachment and institutional trust

Although cognitive value and role perception can directly influence residents’ willingness to participate in age-friendly renovations, the transformation from individual cognition to behavioral willingness still requires certain psychological mechanisms. According to Social Cognitive Theory (SCT), individual behavior is jointly influenced by personal cognition, environmental factors, and psychological perceptions; behavior is not simply a response to environmental stimuli, but rather a process in which individuals actively construct their perception of the environment through their own actions (Bandura, 1986). Therefore, this study further introduces community attachment and institutional trust as mediating variables to explain the internal pathways through which residents’ cognitive factors influence participation willingness.

Community attachment reflects the emotional connection and sense of belonging that residents form with their community, serving as an important psychological foundation for promoting residents’ participation in community public affairs (Wu et al., 2026). When residents develop a strong sense of attachment to their community, they are more inclined to regard community affairs as closely related to themselves, and thus become willing to actively participate. Chang et al. (2021), based on a study of 310 samples from eastern Taiwan, found that place attachment is significantly positively correlated with resident participation. When residents recognize that age-friendly renovations can improve the community environment and enhance overall wellbeing, their emotional investment in community development is further strengthened. Meanwhile, stronger role perception can enhance residents’ awareness of their responsibilities as community members, making them more attentive to collective community interests. Therefore, cognitive value and role perception may promote residents’ participation willingness by strengthening community attachment.

Institutional trust reflects residents’ confidence in the government and community governance bodies regarding policy implementation, fairness, and effectiveness. Ge et al. (2026) found that institutional trust significantly enhances the breadth and depth of residents’ participation in voluntary organizations. Liu et al. (2024b) also confirmed that in China’s top-down governance system, residents’ trust in grassroots government is a key determinant affecting their willingness to participate in old community renewal, and that trust influences participation decisions by shaping residents’ perceived benefits and costs. In the process of age-friendly renovations, residents are concerned not only about the value of the renovation itself but also about whether the implementation process is reliable and whether their rights and interests are protected. According to the Theory of Planned Behavior (TPB), individuals’ judgments about the certainty of behavioral outcomes influence their behavioral willingness. Therefore, higher levels of institutional trust can reduce residents’ uncertainty about the participation process, facilitating the translation of positive cognition into participation behavior.

**FIGURE 1:**
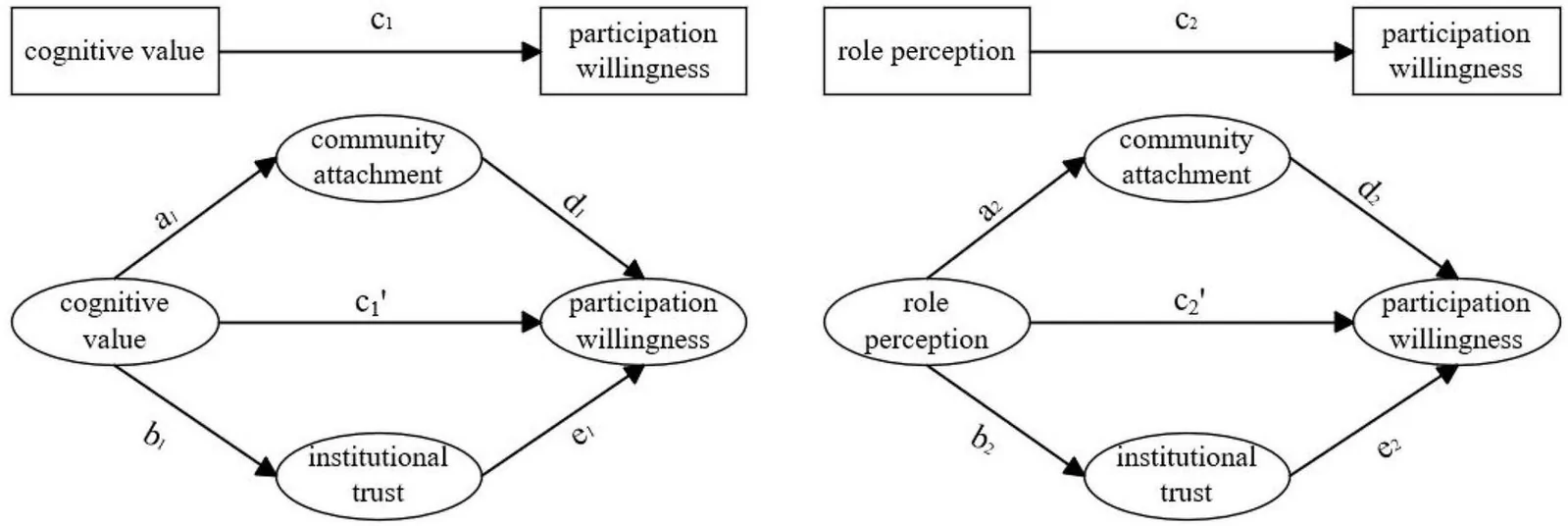
The hypothesized model.

In summary, community attachment and institutional trust function through two dimensions, namely emotional connection and institutional evaluation, jointly constituting important psychological pathways through which cognitive factors influence participation willingness. Accordingly, this study proposes the hypothesis that community attachment and institutional trust mediate the relationships between cognitive value and participation willingness, as well as between role perception and participation willingness. The hypothesized model is shown in Figure 1.

## Materials and methods

### Study design and participants

This study focuses on permanent residents of communities and employs an online cross-sectional survey methodology, selecting multiple representative communities in a city within Jiangsu Province. The sampling employed a combination of stratified random sampling and systematic random sampling: communities were first stratified based on their characteristics and then randomly selected; within each selected community, systematic random sampling was conducted among the permanent residents to determine the final sample population. Jiangsu Province is one of the key pilot regions for the national renovation of aging residential communities and the adaptation of communities for the older adults. Its policy practices demonstrate pioneering and exemplary significance. According to the “Notice on Launching Pilot Demonstration Projects for Age-Friendly Residential Areas” issued by the General Office of the Jiangsu Provincial People’s Government, Jiangsu, as an early pilot region, has actively explored innovative approaches in renovation models, funding mechanisms, and resident participation systems (Jiangsu Provincial People’s Government General Office, 2015). Official data indicates that over the past five years, Jiangsu has cumulatively renovated 6,470 aging residential communities, benefiting 2.052 million households. This represents 125% and 143% completion of the planned targets, respectively, with the renovation tasks for aging communities built before the end of 2000 largely completed (Jiangnan Times Network, 2025). Jiangsu Province has a large stock of aging residential communities with diverse types, including commercial housing, relocation housing, and welfare housing. Convenience sampling enables researchers to delve into specific, representative communities and obtain high-quality, in-depth microdata. Therefore, selecting Jiangsu as the study sample area effectively reflects the practical context and problem characteristics of community age-friendly renovations driven by national policies (Jiangnan Times Network, 2025).

A total of 631 questionnaires were returned, with 595 deemed valid. Current community age-friendly renovations primarily involve collaborative efforts among government, society, and communities, with limited resident participation. Many residents lack awareness of age-friendly renovations. Both the process and outcomes of community age-friendly renovations are intrinsically linked to residents, who are the ultimate beneficiaries and evaluators of these improvements. Therefore, residents should be involved throughout the entire renovation process. Inclusion criteria: (1) Community residents; (2) Age ≥ 18 years; (3) No history of mental illness or psychiatric disorders; (4) Informed consent and voluntary participation in the study. This study strictly adheres to relevant guidelines and regulations. Prior to questionnaire distribution, all participants electronically signed informed consent forms, confirming their understanding of the research content and voluntary participation.

### Measures

Basic sociodemographic information of the participants was collected. The core variables were measured using established scales adapted from previous studies and modified to fit the context of community age-friendly renovations. The measurement sources and specific items for each variable are presented in Table 1.

**TABLE 1:** Variable measurements and item sources.

Variable	Items	Source
Cognitive value	I believe that community age-friendly renovations can improve the overall quality of life in the community.	Adapted from the Perceived Value Scale (PERVAL) developed by Sweeney and Soutar (2001), modified for the context of age-friendly renovation.
	I believe that community age-friendly renovations can bring tangible benefits to residents.	
	I believe that joint participation will make community age-friendly renovations better.	
Role perception	I believe that community age-friendly renovations require active resident participation.	Drawing on the role cognition framework of Biddle (1986) and adapting from the study of Liu et al. (2018), which measured individuals’ perceptions of responsibility and personal roles in public affairs participation.
	I believe that residents are important participants in community age-friendly renovations.	
	I believe that residents have a responsibility to contribute to community age-friendly renovations.	
Community attachment	I feel that I belong to this community.	Adapted from the Sense of Community Theory proposed by McMillan and Chavis (1986) and the Brief Sense of Community Scale (BSCS) developed by Peterson et al. (2008).
	I have a strong sense of belonging to my community.	
	I have emotional connections with other residents in the community.	
	I am willing to contribute my efforts to the development of the community.	
	I believe that the development of the community is closely related to my life.	
Institutional trust	I believe that the community can effectively promote age-friendly renovations.	Adapted from Rieger and Wang (2022), measuring trust in government actions and institutional arrangements
	I believe that the government will ensure the fairness and reasonableness of the age-friendly renovation process.	
	I believe that relevant policies and systems can be effectively implemented.	
	I believe that community staff will seriously respond to residents’ needs.	
Participation willingness	I am willing to participate in community age-friendly renovations.	Adapted from the behavioral intention framework of the Theory of Planned Behavior developed by Ajzen (1991) and the behavioral intention measurement approach of Zeithaml et al. (1996).
	I am willing to attend meetings and discussions related to community age-friendly renovations.	
	I am willing to proactively provide suggestions and opinions for community age-friendly renovations.	
	I am willing to encourage neighbors and family members to participate in community age-friendly renovations.	

#### Assessment of sociodemographic characteristics

Basic information was collected using a standardized questionnaire, including gender, educational level (primary school or below, junior high school, senior high school, college diploma, bachelor’s degree or above), monthly income (≤ 3,000, 3,001–4,000, 4,001–5,000, 5,001–6,000, ≥ 6,001 RMB), self-rated health (poor, fair, good), and housing type (self-built on homestead land, demolition compensation housing, commercial housing, welfare housing, affordable housing, business apartments, industrial factory dormitories).

#### Cognitive value and role perception

Cognitive value refers to residents’ overall perception of the benefits generated by community age-friendly renovations. This study adapted the Perceived Value Scale developed by Sweeney and Soutar (2001) to the public service context, with 3 items established. Role perception refers to residents’ cognition of their roles, responsibilities, and obligations in the renovation process. Drawing on the role cognition framework of Biddle (1986) and the study of Liu et al. (2018), we contextualized the original scales to suit community residents’ participation in public affairs, with 3 items established. All items were measured on a 5-point Likert scale (1 = Strongly disagree, 5 = Strongly agree). The Cronbach’s α coefficients for cognitive value and role perception were 0.914 and 0.851, respectively, indicating good reliability.

#### Community attachment and institutional trust

Community attachment refers to the emotional connection, identity, and psychological attachment that residents form with their community. Drawing on the Sense of Community Theory of McMillan and Chavis (1986) and the Brief Sense of Community Scale (BSCS) of Peterson et al. (2008), we adapted the measurement items to the context of community age-friendly renovations, with 5 items established to reflect residents’ sense of identity, belonging, and emotional connection. Institutional trust refers to residents’ trust in the government, community organizations, and relevant institutional arrangements, reflecting their recognition of governance competence, policy implementation, and institutional effectiveness. Adapting the measurement of government trust from Rieger and Wang (2022) to the Chinese age-friendly renovation context, we established 4 items. All items were measured on a 5-point Likert scale (1 = Strongly disagree, 5 = Strongly agree). The Cronbach’s α coefficients for community attachment and institutional trust were 0.897 and 0.967, respectively, indicating good reliability.

#### Participation willingness

Participation willingness refers to residents’ subjective willingness and behavioral tendency to participate in activities related to community age-friendly renovations in the future. This study adapted the measurement framework of “behavioral intention” from the Theory of Planned Behavior (Ajzen, 1991), drew upon the multidimensional measurement approach to behavioral intentions developed by Zeithaml et al. (1996), and contextualized the scale according to the community governance context of age-friendly renovations. A total of 4 items were established, all of which were measured using a 5-point Likert scale (1 = Strongly disagree, 5 = Strongly agree). The Cronbach’s α coefficient for participation willingness was 0.962, indicating good reliability.

### Data analysis

All statistical analyses were performed using SPSS 26.0 and the PROCESS macro v4.1 for SPSS.

Reliability and validity: Cronbach’s α coefficient was used to test the internal consistency reliability of each scale. Following Fornell and Larcker (1981), composite reliability (CR) and average variance extracted (AVE) were calculated to assess the validity of the measurement model.

Descriptive and preliminary analyses: Continuous variables following a normal distribution were expressed as mean ± standard deviation (SD), while categorical variables (e.g., gender, education level, monthly income) were presented as frequencies and percentages (*N*%). One-way analysis of variance (ANOVA) and independent samples *t*-tests were employed to examine differences in participation willingness across sociodemographic groups. Pearson correlation analysis was conducted to explore bivariate relationships among cognitive value, role perception, community attachment, institutional trust, and participation willingness. To identify significant predictors of participation willingness, multiple linear regression analysis was performed. Prior to regression, multicollinearity diagnostics were run using variance inflation factor (VIF) for all independent variables.

Mediation analysis: The hypothesized mediation model (Figure 1) was tested using the PROCESS macro v4.1 for SPSS (Model 4), a widely used tool for assessing mediating effects in contemporary research (Hayes, 2018). In this model, cognitive value and role perception were specified as predictor variables, community attachment and institutional trust as mediators, and participation willingness in age-friendly renovations as the dependent variable. Sociodemographic characteristics including gender, self-rated health, and housing type were entered as covariates to control for potential confounding effects.

Specifically, coefficients c_1_′ and c_2_′ represent the direct effects of cognitive value and role perception on participation willingness, respectively. The indirect effects were computed as products of path coefficients: a_1_ × d_1_ (cognitive value → community attachment → participation willingness) and b_1_ × e_1_ (cognitive value → institutional trust → participation willingness); similarly, a_2_ × d_2_ (role perception → community attachment → participation willingness) and b_2_ × e_2_ (role perception → institutional trust → participation willingness). The total effects of cognitive value (c_1_) and role perception (c_2_) on participation willingness are decomposed as c_1_ = c_1_′ + a_1_ × d_1_ + b_1_ × e_1_ and c_2_ = c_2_′ + a_2_ × d_2_ + b_2_ × e_2_.

Bootstrapping with 5,000 resamples was used to generate point estimates and 95% confidence intervals for all indirect effects. The indirect effect was considered statistically significant if its corresponding 95% CI did not include zero. All statistical tests were two-tailed, with significance set at α = 0.05.

## Results

### Differences in participation willingness for age-friendly renovations among community residents with diverse sociodemographic characteristics

This study employed a descriptive quantitative research design, wherein residents from several typical communities in a city of Jiangsu Province were selected as research subjects through random sampling with self-administered questionnaires. A total of 631 questionnaires were distributed, among which 595 valid ones were retrieved, yielding a valid response rate of 94.29%. Among the participants, 246 were male (41.3%) and 349 were female (58.7%). Ages ranged from 18 to 70 years, with an average age of (49.7 ± 7.1) years. To ensure data quality, 36 invalid questionnaires were excluded, including those with inconsistent responses or completion times under 100 s. According to American sociologist Babbie (2005), a response rate ≥ 60% in social science surveys meets general requirements, while ≥ 70% is considered excellent. The questionnaire response rate in this study meets the minimum standard for social science surveys, allowing for normal data analysis.

Univariate analysis revealed no statistically significant differences in participation willingness across different sociodemographic groups (all *P*-values > 0.05) (Table 2). A comprehensive analysis of sociodemographic characteristics revealed that while minor mean differences existed between some groups, none reached statistical significance. This finding indicates that community residents generally maintain moderately positive attitudes toward age-friendly renovations (overall mean≈3.23), and traditional demographic variables are not key predictors of participation willingness. Future research should focus more on underlying motivational factors to more effectively advance the implementation of age-friendly renovation policies.

**TABLE 2:** Participation willingness of community residents in age-friendly renovations by different sociodemographic characteristics (*N* = 595).

Characteristics	Categories	*N* (%)	Participation willingness		
			Mean ± SD	*t* or *F*	*P*-value
Gender	Male	246 (41.3)	3.15 ± 1.34	−1.318	0.188
	Female	349 (58.7)	3.30 ± 1.26		
Age	18–30	0	0	0.095	0.963
	31–40	41 (6.9)	3.28 ± 1.33		
	41–50	300 (50.4)	3.22 ± 1.29		
	51–60	219 (36.8)	3.23 ± 1.29		
	61–70	35 (5.9)	3.34 ± 1.34		
Education level	Primary or below	62 (10.4)	3.06 ± 1.27	1.101	0.355
	Junior high	211 (35.5)	3.23 ± 1.30		
	Senior high	157 (26.4)	3.39 ± 1.23		
	College diploma	127 (21.3)	3.20 ± 1.35		
	Bachelor’s or above	38 (6.4)	3.04 ± 1.35		
Income	3,000 or below	34 (5.7)	3.26 ± 1.24	0.754	0.556
	3,001–4,000	130 (21.8)	3.20 ± 1.35		
	4,001–5,000	163 (27.4)	3.36 ± 1.25		
	5,001–6,000	212 (35.6)	3.22 ± 1.30		
	6,001 and above	56 (9.4)	3.03 ± 1.27		
Self-rated health	Poor	172 (28.9)	3.39 ± 1.23	1.65	0.193
	Fair	197 (33.1)	3.16 ± 1.34		
	Good	226 (38.0)	3.19 ± 1.29		
Housing type	Self-built on homestead land	136 (22.9)	3.15 ± 1.33	0.831	0.546
	Demolition compensation housing	147 (24.7)	3.32 ± 1.23		
	Commercial housing	162 (27.2)	3.34 ± 1.27		
	Welfare housing	76 (12.8)	3.01 ± 1.42		
	Affordable housing	38 (6.4)	3.14 ± 1.24		
	Business apartments	24 (4.0)	3.35 ± 1.29		
	Industrial factory dormitories	12 (2.0)	3.27 ± 1.26		

SD, standard deviation.

### Reliability and validity analysis

This study employed Cronbach’s α coefficient, composite reliability (CR), and average variance extracted (AVE), along with the Fornell–Larcker criterion, to assess the reliability and validity of the measurement model. As shown in Table 3, the CR values for all latent variables ranged from 0.852 to 0.965, all exceeding the recommended threshold of 0.70, indicating good internal consistency reliability. Although the CR values for institutional trust and participation willingness exceeded 0.95, all items were retained because they capture different theoretical aspects of their respective constructs and demonstrate high factor loadings. The high CR values reflect good internal consistency rather than item redundancy. The AVE values all surpassed the recommended threshold of 0.50, demonstrating satisfactory convergent validity. Discriminant validity was further assessed using the Fornell–Larcker criterion. The square roots of the AVE for each latent variable (diagonal values) were all greater than the correlations between that variable and any other variable, confirming adequate discriminant validity among the constructs.

**TABLE 3:** Reliability and validity of the measurement model.

Variable	Cronbach’s α	AVE	CR	Cognitive value	Role perception	Community attachment	Institutional trust	Participation willingness
Cognitive value	0.914	0.800	0.922	**0.896**	–	–	–	–
Role perception	0.851	0.658	0.852	0.798	**0.800**	–	–	–
Community attachment	0.897	0.637	0.898	0.302	0.314	**0.795**	–	–
Institutional trust	0.967	0.874	0.965	0.087	0.137	0.136	**0.933**	–
Participation willingness	0.962	0.856	0.959	0.207	0.229	0.228	0.465	**0.925**

Bold values represent the square root of AVE for each construct.

### Scores of the questionnaire

The questionnaire employed a 5-point Likert scale (1 = “Strongly Disagree,” 2 = “Disagree,” 3 = “Neutral,” 4 = “Agree,” 5 = “Strongly Agree”). The overall average score for age-friendly renovation willingness among 595 community residents was 31.86 ± 9.22, with an average score of 3.19 ± 0.92 across all items. Specifically, the mean score for the cognitive value dimension was 3.15 ± 1.19, the role perception dimension was 3.16 ± 1.09, and the participation willingness dimension was 3.24 ± 1.29 (Table 4).

**TABLE 4:** Questionnaire scores for cognitive value, role perception, and participation willingness (M ± SD).

Variable	Minimum	Maximum	Score	Average score of items
Cognitive value	3	15	9.44 ± 3.57	3.15 ± 1.19
Role perception	3	15	9.48 ± 3.28	3.16 ± 1.09
Participation willingness	4	20	12.95 ± 5.17	3.24 ± 1.29
Total participation willingness score	11	49	31.86 ± 9.22	3.19 ± 0.92

### Correlations among cognitive value, role perception, community attachment, institutional trust, and participation willingness

Pearson correlation analysis was conducted to examine the bivariate relationships among the core study variables. As shown in Table 5, cognitive value, role perception, community attachment, institutional trust, and participation willingness were all positively correlated with each other, with all correlation coefficients significantly greater than zero. Among these, institutional trust showed the strongest correlation with participation willingness (*r* = 0.465, *p* < 0.001). These results provide preliminary support for the subsequent regression and mediation analyses.

**TABLE 5:** Pearson correlations among cognitive value, role perception, community attachment, institutional trust, and participation willingness (*N* = 595).

Variable	Mean ± SD	Cognitive value	Role perception	Community attachment	Institutional trust	Participation willingness
Cognitive value	9.44 ± 3.57	1.000	–	–	–	–
Role perception	9.48 ± 3.28	0.798***	1.000	–	–	–
Community attachment	16.20 ± 5.20	0.302***	0.314***	1.000	–	–
Institutional trust	12.60 ± 5.48	0.087*	0.137**	0.136**	1.000	–
Participation willingness	12.95 ± 5.17	0.207***	0.229***	0.228***	0.465***	1.000

SD, standard deviation.

*p < 0.05;

**p < 0.01;

***p < 0.001.

### Testing the mediating effects of community attachment and institutional trust

To ensure the robustness of the multiple linear regression and mediation analyses, multicollinearity diagnostics were first conducted. In the model with cognitive value as the independent variable, the variance inflation factor (VIF) values for cognitive value, community attachment, and institutional trust were 1.112, 1.111, and 1.025, respectively. In the model with role perception as the independent variable, the VIF values for role perception, community attachment, and institutional trust were 1.133, 1.116, and 1.037, respectively. Tolerance values for all variables were close to 1, and all VIF values were well below the conservative threshold of 5. These results indicate that, after controlling for other variables, the predictor variables exhibit good independence with no severe multicollinearity issues (Stine, 1995), ensuring the stability of the subsequent regression and mediation analyses.

**FIGURE 2:**
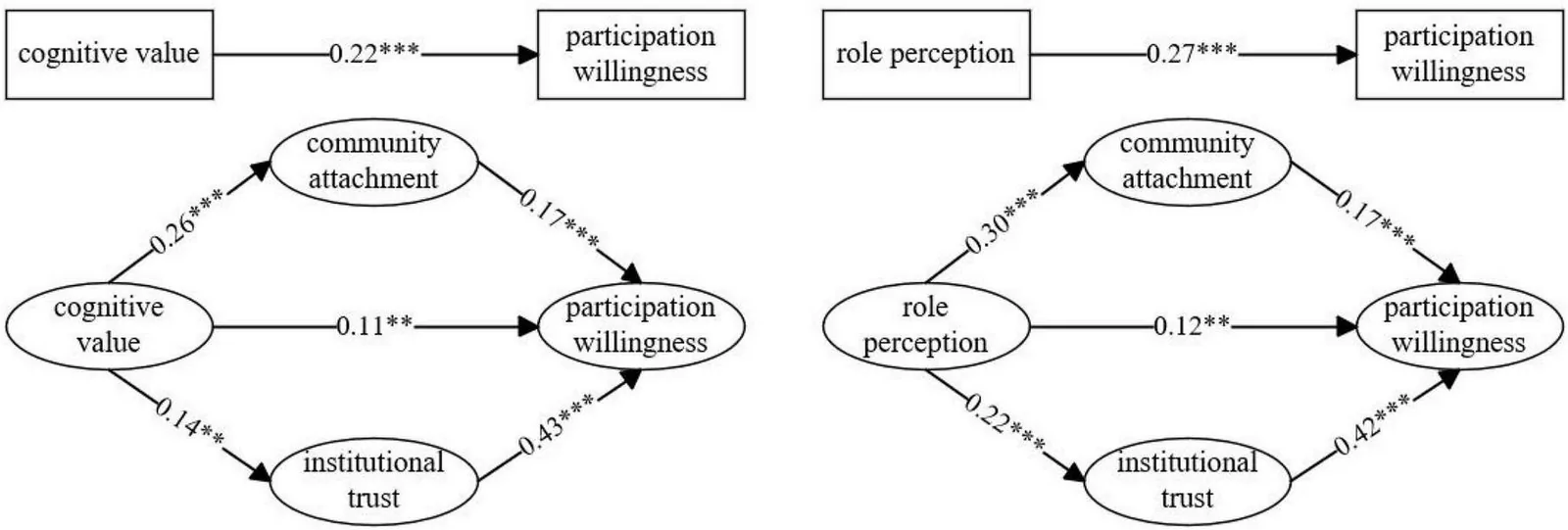
Path diagram of variables. ***P < 0.001; **P < 0.01.

Mediation analyses were conducted using Model 4 of the PROCESS macro for SPSS (version 4.1) to examine the mediating roles of community attachment and institutional trust in the relationships between (a) cognitive value and participation willingness, and (b) role perception and participation willingness. The results are presented in Tables 6, 7 and illustrated in Figure 2.

**TABLE 6:** Mediation model results for cognitive value, community attachment, institutional trust, and participation willingness.

Variables	Model 1 (participation willingness)			Model 2 (community attachment)			Model 3 (institutional trust)			Model 4 (participation willingness)		
	B (SE)	*t*	*P*-value	B (SE)	*t*	*P*-value	B (SE)	*t*	*P*-value	B (SE)	*t*	*P*-value
Age	0.020 (0.074)	0.275	0.784	0.055 (0.058)	0.947	0.344	−0.007 (0.080)	−0.088	0.930	0.014 (0.064)	0.214	0.830
Education level	−0.121 (0.123)	−0.982	0.326	0.111 (0.097)	1.148	0.251	0.014 (0.133)	0.102	0.919	−0.146 (0.108)	−1.359	0.175
Gender	0.208 (0.118)	1.774	0.077	−0.007 (0.092)	−0.074	0.941	0.117 (0.127)	0.926	0.355	0.159 (0.102)	1.555	0.12
Self-rated health	−0.103 (0.076)	−1.354	0.176	0.042 (0.060)	0.699	0.485	−0.193 (0.082)	−2.369	0.018	−0.027 (0.066)	−0.404	0.686
Housing type	−0.016 (0.044)	−0.362	0.717	0.021 (0.035)	0.59	0.556	−0.046 (0.048)	−0.97	0.333	0.000 (0.039)	0.006	0.995
Income	−0.141 (0.117)	−1.201	0.23	0.034 (0.092)	0.364	0.716	0.054 (0.126)	0.431	0.667	−0.170 (0.102)	−1.664	0.097
Cognitive value	0.221 (0.044)	5.02	*P* < 0.001	0.265 (0.035)	7.669	*P* < 0.01	0.144 (0.047)	3.038	*P* < 0.01	0.113 (0.040)	2.806	*P* < 0.01
Community attachment	–	–	–	–	–	–	–	–	–	0.173 (0.046)	3.752	*P* < 0.001
Institutional trust	–	–	–	–	–	–	–	–	–	0.429 (0.034)	12.801	*P* < 0.001
*R* ^2^	0.053	–	–	0.103	–	–	0.028	–	–	0.284	–	–
*F*	4.731 (*P* < 0.001)	–	–	9.654 (*P* < 0.001)	–	–	2.453 (*P* < 0.05)	–	–	25.813 (*P* < 0.001)	–	–
Total effect of CV (X) on PW (Y)												
		Coeff	Bootstrap SE	95% Bootstrap CI								
		0.221	0.044	(0.134, 0.307)								
Indirect effect: CV (X) → CA/IT (MED) → PW (Y)												
		Coeff	Bootstrap SE	95% Bootstrap CI								
		0.108	0.026	(0.057, 0.158)								
Direct effect: CV (X)→ PW (Y)												
		Coeff	Bootstrap SE	95% Bootstrap CI								
		0.113	0.04	(0.034, 0.193)								

B, unstandardized regression coefficient; SE, standard errors. Coeff, coefficient; SE, standard errors; CI, confidence interval; X, independent variable; Y, dependent variable; M, mediator. Mediator variables include community attachment and institutional trust.

**TABLE 7:** Mediation model results for role perception, community attachment, institutional trust, and participation willingness.

Variables	Model 5 (participation willingness)			Model 6 (community attachment)			Model 7 (institutional trust)			Model 8 (participation willingness)		
	B (SE)	*t*	*P*-value	B (SE)	*t*	*P*-value	B (SE)	*t*	*P*-value	B (SE)	*t*	*P*-value
Age	0.008 (0.073)	0.105	0.917	0.040 (0.058)	0.684	0.494	−0.015 (0.079)	−0.187	0.852	0.007 (0.064)	0.111	0.912
Education level	−0.127 (0.123)	−1.034	0.302	0.101 (0.097)	1.048	0.295	0.016 (0.132)	0.121	0.904	−0.151 (0.108)	−1.403	0.161
Gender	0.236 (0.117)	2.015	0.044	0.022 (0.092)	0.239	0.811	0.142 (0.126)	1.129	0.259	0.172 (0.103)	1.675	0.095
Self-rated health	−0.118 (0.075)	−1.569	0.117	0.022 (0.059)	0.365	0.715	−0.201 (0.081)	−2.483	0.013	−0.037 (0.066)	−0.553	0.581
Housing type	−0.015 (0.044)	−0.33	0.742	0.023 (0.035)	0.658	0.511	−0.046 (0.047)	−0.972	0.331	0.001 (0.039)	0.027	0.978
Income	−0.140 (0.116)	−1.205	0.229	0.030 (0.092)	0.326	0.745	0.062 (0.125)	0.493	0.622	−0.171 (0.102)	−1.682	0.093
Role perception	0.269 (0.048)	5.657	*P* < 0.001	0.296 (0.038)	7.887	*P* < 0.001	0.222 (0.051)	4.347	*P* < 0.001	0.124 (0.044)	2.8	*P* < 0.01
Community attachment	–	–	–	–	–	–	–	–	–	0.173 (0.046)	3.738	*P* < 0.001
Institutional trust	–	–	–	–	–	–	–	–	–	0.429 (0.034)	12.564	*P* < 0.001
*R* ^2^	0.064	–	–	0.108	–	–	0.044	–	–	0.284		
*F*	5.718 (*P* < 0.001)	–	–	10.146 (*P* < 0.001)	–	–	3.852 (*P* < 0.001)	–	–	25.808 (*P* < 0.001)		
Total effect of RP (X) on PW (Y)												
		Coeff	Bootstrap SE	95% Bootstrap CI								
		0.269	0.048	(0.176, 0.363)								
Indirect effect: RP (X) → CA/IT (MED) → PW (Y)												
		Coeff	Bootstrap SE	95% Bootstrap CI								
		0.145	0.028	(0.093, 0.201)								
Direct effect: RP (X)→ PW (Y)												
		Coeff	Bootstrap SE	95% Bootstrap CI								
		0.124	0.044	(0.037, 0.211)								

B, unstandardized regression coefficient; SE, standard errors. Coeff, coefficient; SE, standard errors; CI, confidence interval; X, independent variable; Y, dependent variable; M, mediator. Mediator variables include community attachment and institutional trust.

Multiple linear regression results: Multiple linear regression analysis revealed that cognitive value, role perception, community attachment, and institutional trust were the primary significant predictors of participation willingness (*F* = 25.81, *P* < 0.001, *R*^2^ = 0.284), whereas control variables (e.g., age, education level) were not significant in any of the models (all *P* > 0.05). This suggests that the core theoretical variables of this study are more critical in shaping residents’ participation willingness than sociodemographic characteristics, whose limited independent effects may be attributable to unmeasured confounding factors.

Total and direct effects: After controlling for sociodemographic factors, the total effect of cognitive value on participation willingness was significant (c_1_ = 0.221, *P* < 0.001), as was the total effect of role perception (c_2_ = 0.269, *P* < 0.001). After introducing community attachment and institutional trust as mediators into the model, the direct effects of both cognitive value and role perception on participation willingness remained significant (c_1_′ = 0.113, *P* < 0.001; c_2_′ = 0.124, *P* < 0.01).

Path coefficients: Cognitive value significantly predicted both mediators: community attachment (a_1_ = 0.296, *P* < 0.001) and institutional trust (b_1_ = 0.144, *P* < 0.01). Similarly, role perception significantly predicted community attachment (a_2_ = 0.265, *P* < 0.001) and institutional trust (b_2_ = 0.222, *P* < 0.001). Both mediators, in turn, significantly predicted participation willingness (for the cognitive value model: d_1_ = 0.173, *P* < 0.001; e_1_ = 0.429, *P* < 0.001; for the role perception model: d_2_ = 0.172, *P* < 0.001; e_2_ = 0.424, *P* < 0.001).

Indirect effects: To evaluate the indirect effects, 5,000 Bootstrap resamples were drawn from the original dataset. The total indirect effect of cognitive value on participation willingness through community attachment and institutional trust was a_1_ × d_1_ + b_1_ × e_1_ = 0.108, with a 95% Bootstrap confidence interval (CI) ranging from 0.057 to 0.158. The total indirect effect of role perception on participation willingness was a_2_ × d_2_ + b_2_ × e_2_ = 0.145, with a 95% Bootstrap CI ranging from 0.093 to 0.201. Both confidence intervals excluded zero, indicating that the mediating effects were statistically significant (Zhou et al., 2025).

Summary of mediation: In summary, community attachment and institutional trust play significant mediating roles in both the relationship between cognitive value and participation willingness and the relationship between role perception and participation willingness. The final model accounted for 28.4% of the total variance in participation willingness (*R*^2^ = 0.284).

## Discussion

Against the backdrop of national policies promoting old community renovation and age-friendly society construction (Zhang and Loukaitou-Sideris, 2025), age-friendly community renovation has emerged as a key strategy to address population aging. As both direct participants in renovation services and co-beneficiaries of future eldercare transformation, residents’ collective attitudes and behaviors significantly shape the sustainability of community eldercare services and the basis for intergenerational harmony.

Univariate analysis revealed no statistically significant differences in residents’ willingness to participate in age-friendly renovations across sociodemographic characteristics such as gender, age, and educational level (all *P* > 0.05), indicating that individual background factors did not exert a substantial influence on participation willingness. This finding aligns with Sun and Sun (2024), suggesting that value recognition of age-friendly renovations, as a universal public welfare initiative, may have surpassed individual demographic differences; participation willingness stems more from universal psychological mechanisms independent of demographic variables, such as individuals’ perception of issue relevance, expectations of behavioral outcomes, and collective efficacy. Further analysis showed that the overall mean score of participation willingness was 3.23 (moderately high), indicating that residents hold positive attitudes but have not yet translated them into strong behavioral intentions, which aligns with the cognitive deficit dilemma noted by Zhou and Chen (2022). These findings suggest that policy-making should focus on two key aspects: strengthening policy publicity to enhance residents’ awareness of renovation benefits, and establishing community consultation and co-governance mechanisms to transform general recognition into effective participation. Correlation and regression analyses indicated that cognitive value, role perception, community attachment, and institutional trust were all significant positive predictors of participation willingness, supporting the proposed hypotheses and aligning with the findings of Helbich (2018) that the built environment influences health-related behaviors. Bootstrap mediation tests further validated the second hypothesis, cognitive value and role perception not only exerted significant direct positive effects on participation willingness but also produced significant indirect effects through the parallel mediating pathways of community attachment and institutional trust, revealing a dual-drive mechanism underlying residents’ participation willingness. This finding is consistent with the triadic reciprocal determinism model of Social Cognitive Theory (Bandura, 1986; Le et al., 2026), in which individual cognition drives behavior, and behavioral outcomes in turn reinforce or modify perceptions, providing a systematic theoretical explanation for understanding residents’ decision-making in public affairs participation.

The core theoretical contribution of this study lies in identifying and validating a dual mediation model of community attachment and institutional trust, overcoming the limitations of previous studies that adopted a single-perspective approach. The model reveals that the successful implementation of age-friendly renovations depends on the activation of social capital and the establishment of institutional legitimacy: residents’ perceived community attachment promotes participation through peer influence and normative pressure, fostering individual trust in the measures (Yue et al., 2025), while institutional trust enhances residents’ confidence by reducing concerns about policy uncertainty (Musso and Weare, 2015). Together, these two factors explain why the core independent variables still exert a significant influence on participation willingness after controlling for demographic variables. Accordingly, promoting age-friendly renovations requires cultivating neighborhood mutual-aid networks and building government credibility, thereby operationalizing the constructs of subjective norms and perceived behavioral control from the Theory of Planned Behavior within the Chinese community governance context. Institutional trust demonstrated the strongest mediating effect in this study, which should be interpreted within the governance model characterized by government leadership, social collaboration, and resident participation (Central Committee of the Communist Party of China, and State Council of the People’s Republic of China, 2017; Ge et al., 2026). Its powerful mediating role stems from multiple factors: as a typical livelihood project, age-friendly renovations rely heavily on the administrative system, and high trust reduces concerns about policy uncertainty (Zeng et al., 2024); resident participation transcends individual short-term interests, reflecting deeper expectations for the future eldercare system (Liu and Liu, 2026); internalized collectivist values lead residents to view community affairs as collective responsibilities (Huang and Yu, 2022), and institutional trust reconstructs the cognitive basis of solidarity through procedural fairness, curbing free-riding tendencies. The stronger effect of institutional trust over community attachment can be attributed to the fact that, because livelihood projects are government-led and residents care most about government directives, residents perceive participation as safe and reliable only when they trust the government (Zhang et al., 2022); community attachment, in contrast, fails to address fundamental concerns such as whether policies will change. Social Cognitive Theory (Tan et al., 2025) further explains this mechanism, the physical environment reduces concerns about the complexity of participation by enhancing institutional trust as a psychological mediator, thereby translating environmental needs into participation willingness.

Although the present study validated the significant effects of cognitive value, role perception, community attachment, and institutional trust on residents’ willingness to participate in age-friendly renovations, the structural model explained approximately 28% of the variance in participation willingness. This suggests that while the above psychological and social relational factors account for a substantial portion of residents’ participation decisions, other influential factors not incorporated into the model remain to be identified. As a collective action involving public interests, community governance, and resource allocation, participation in age-friendly renovations may also be shaped by broader social and contextual factors. For instance, from the perspective of the social environment, neighborhood interaction, community social capital, and group norms may influence residents’ participation tendencies through social influence mechanisms. From the perspective of the institutional environment, policy implementation effectiveness, accessibility of information, perceived procedural fairness, and the level of government support may further affect residents’ trust in renovation projects and their willingness to participate. From the perspective of individual characteristics, factors such as prior participation experience, health status, economic conditions, and digital information access capability may also contribute to variations in participation willingness across different residents. Therefore, future research could further integrate multidimensional factors from psychological, social, and institutional domains to construct a more comprehensive explanatory framework for residents’ participation willingness.

Furthermore, the findings of this study should be understood in the broader context of international aging research. The World Health Organization, grounded in the concept of active aging, emphasizes that continuous social participation of older adults is an essential component of healthy, active, and dignified aging (World Health Organization [WHO], 2002), and that a supportive community environment and social support can facilitate older adults’ engagement in community activities and public affairs. In one New Zealand study, Wiles et al. (2012) found that community attachment, sense of safety, and emotional connections with the living environment are important foundations for older adults’ choice to age in place, suggesting that community attachment is not merely an emotional bond but also a crucial psychological resource that promotes older adults’ active participation in community affairs (Wiles et al., 2012). Research from Norway indicates that institutional trust is closely associated with residents’ participation in local governance, and trust in local institutions and governing bodies is an important condition for effective public participation (Holum, 2023). Studies from the United States on older adults’ participation in age-friendly community development have found that their engagement stems not only from personal benefit considerations but also from a sense of social contribution, community connectedness, and self-fulfillment (Cao et al., 2024). Therefore, the pathways identified in this study based on the Chinese community context are consistent with international research findings on active aging and community participation, while also reflecting the significant influence of community relationships and institutions on residents’ participatory behavior.

This study carries both theoretical and practical significance for deepening the implementation of community age-friendly renovations and optimizing related policies. Theoretically, based on the formation mechanism of participation willingness, this study not only empirically confirms the core driving roles of cognitive value and role perception, but also transcends the singular explanatory pathways of environment to behavior or cognition to behavior. By introducing community attachment and institutional trust as parallel mediators, it constructs and validates a dual-path model, offering a novel systemic perspective for understanding residents’ decision-making in public affairs. This framework guides future intervention strategies to shift from mere physical environment improvement toward the coordinated cultivation of social capital and institutional trust. Practically, government departments and community practitioners should adopt a coordinated approach across three levels. At the cognitive level, transparent policy communication and community hearings should be used to strengthen residents’ cognitive value and sense of participatory role. At the social level, building-level mutual aid groups and community activities should be organized to cultivate neighborhood networks and enhance community attachment. At the institutional level, open and fair renovation procedures should be established to embed trust-building throughout the entire project cycle, ultimately enhancing residents’ willingness to participate.

Several limitations should be acknowledged in this study. First, the use of convenience sampling may have introduced selection bias, which could affect the representativeness and external validity of the findings. Residents who were more willing to participate in the survey may differ from non-participants in terms of health awareness, previous community engagement, or perceptions of age-friendly renovation, potentially influencing the estimation of participation willingness. Future studies should consider adopting multistage stratified random sampling methods with larger and more geographically diverse samples to improve representativeness and enhance the generalizability of the findings. Second, the cross-sectional design limits the ability to establish causal relationships among variables. Although the mediation analysis provided insights into the potential mechanisms underlying residents’ participation willingness, the temporal relationships between predictors, mediators, and outcomes could not be confirmed. Therefore, reverse causality and unmeasured confounding factors cannot be fully excluded. Longitudinal studies or intervention-based designs are warranted to further validate the proposed causal pathways. Moreover, given variations in socioeconomic conditions, cultural contexts, and community governance across regions, the findings should be interpreted cautiously when applied to other settings. Future research involving larger-scale, cross-regional comparisons may further improve the generalizability of the results. Third, the self-developed questionnaire may have certain measurement limitations. As the data were self-reported, residents’ responses may have been influenced by social desirability bias, potentially affecting the accuracy of the measured outcomes. Future studies could integrate objective behavioral indicators, community participation records, or qualitative approaches to provide a more comprehensive assessment of residents’ participation intentions and behaviors. Finally, residents’ willingness to participate in community age-friendly renovation is a complex decision-making process influenced by multiple individual, social, and contextual factors. This study did not include all potential determinants. Future research could incorporate additional variables, such as previous community participation experiences, social support, community belonging, and institutional trust, to develop a more comprehensive theoretical framework and further explore the direct and indirect pathways influencing residents’ participation willingness.

## Conclusion

This study developed and validated a mediation model aimed at exploring the mechanisms through which community attachment and institutional trust mediate the formation of community residents’ participation willingness in age-friendly renovations. The results indicate that cognitive value and role perception not only exert a direct and significant positive influence on participation willingness but also indirectly affect willingness through the partial mediating roles of community attachment and institutional trust. From the psychological perspective, cognitive value and role perception form the cognitive foundation of participation motivation. When residents recognize the positive benefits of the renovation and internalize participation as their personal responsibility, behavioral intention gains an intrinsic driving force. The mediating role of community belongingness demonstrates how satisfaction with this sense of belonging promotes behavioral change, whereas the mediating role of institutional trust reflects residents’ psychological sense of security regarding the policy environment, low trust may trigger psychological defenses, thereby inhibiting participation. Both approaches jointly explain how cognitive factors translate into participation willingness through social context perception, operating along two pathways: emotional connection and institutional trust.

In summary, communities and relevant departments may implement coordinated intervention strategies that combine cognitive empowerment and environmental optimization to systematically enhance residents’ participation willingness. Specific measures include integrating awareness of the value of age-friendly renovations and guidance on residents’ roles into community education and services, as well as designing public activities that strengthen community cohesion and institutional trust; incorporating residents’ cognitive value and role perception into community governance evaluation systems, and formulating participation guidelines and incentive measures for age-friendly renovations; and supporting grassroots communities in implementing evidence-based participation promotion programs, thereby achieving deep integration of cognitive enhancement and the development of supportive social environments, and laying the foundation for building healthy and livable environments for middle-aged and older adults.

## Data Availability

The original contributions presented in this study are included in this article/supplementary material, further inquiries can be directed to the corresponding authors.
